# Hepatic Glycerol Metabolism-Related Genes in Carnivorous Rainbow Trout (*Oncorhynchus mykiss*): Insights Into Molecular Characteristics, Ontogenesis, and Nutritional Regulation

**DOI:** 10.3389/fphys.2020.00882

**Published:** 2020-07-31

**Authors:** Stephane Panserat, Elisabeth Plagnes-Juan, Elsa Gazzola, Mariana Palma, Leonardo J. Magnoni, Lucie Marandel, Ivan Viegas

**Affiliations:** ^1^INRAE, Université de Pau et des Pays de l’Adour, E2S UPPA, NuMéA, Saint-Pée-sur-Nivelle, France; ^2^Centre for Functional Ecology, Department of Life Sciences, University of Coimbra, Coimbra, Portugal; ^3^Interdisciplinary Centre of Marine and Environmental Research (CIIMAR), University of Porto, Matosinhos, Portugal; ^4^Centre for Neuroscience and Cell Biology, University of Coimbra, Coimbra, Portugal

**Keywords:** glycerol metabolism, fish nutrition, genomics, gene expression, rainbow trout, aquaculture

## Abstract

Glycerol metabolism in rainbow trout is poorly studied even though it is at the interface between lipid and glucose metabolism. Moreover, glycerol can be an important ingredient in new aquafeed formulation to decrease the catabolism of dietary amino acids. Thus, the present study aimed to characterize for the first time the different genes coding for key enzymes and proteins involved in hepatic glycerol metabolism. From the trout genomes, all the paralogous genes coding for glycerol transport (*aqp9b*), glycerol kinase (*gk2a* and *gk5*), glycerol-3-phosphate phosphatase (*pgp*), and glycerol-3-phosphate dehydrogenase (*gpd1a*, *gpd1b*, and *gpd1c*) were identified. The ontogenesis determined that the capacity to metabolize glycerol begins with the apparition of the liver during the development (stage 22) and are more expressed at the endogenous–exogenous feeding period (stage 35). The postprandial regulation of the expression of these genes in juvenile trout showed that the postprandial peak of expression is between 4 and 24 h after the last meal for many of the genes, demonstrating that glycerol metabolism could be nutritionally regulated at a molecular level. However, surprisingly, no regulation of the mRNA abundance for the glycerol metabolism-related genes by different levels of dietary glycerol (0, 2.5, and 5%) have been detected, showing that hepatic glycerol metabolism is poorly regulated at a molecular level by dietary glycerol in rainbow trout juveniles.

## Introduction

The production of aquafeeds based on fishmeal and fish oils has been a key element in recent aquaculture development (Klinger and Naylor, 2012; Food Agric Organ, 2018). However, the stability of fishmeal and fish oil production and the dramatic increase in their costs, as well as the challenge of improving growth while reducing these production costs, are compromising the sustainable development of aquaculture. Fish diets have evolved to include an increased proportion of plant protein sources and vegetable oils (Gatlin et al., 2007; Naylor et al., 2009; Food Agric Organ, 2018). Unfortunately, no adequate complete replacement for fishmeal and fish oil, with no negative effects on growth performance and fish quality, has yet been found (Panserat et al., 2009; Lazzarotto et al., 2018). Moreover, prices for grains and vegetable oils have increased, forcing the livestock industry to look for alternative feedstuffs. Among them, glycerol, a widely available and inexpensive source of dietary energy issue as a coproduct of biodiesel production (Ayoub and Abdullah, 2012; Quispe et al., 2013; Tan et al., 2013), has been proposed as a dietary component to replace other ingredients included in feeds.

The inclusion of glycerol in aquafeeds has been mainly investigated in the omnivorous fish Nile tilapia (*Oreochromis niloticus*), showing that growth performance, voluntary feed intake, and protein efficiency could be affected depending on the inclusion level (Gonçalves et al., 2015). However, low inclusion levels of glycerol in this species (3–6%) did not alter growth performance, protein efficiency ratio, survival rate, lipid deposition, or hepatosomatic and viscerosomatic indexes compared to fish fed a diet without glycerol (Meurer et al., 2012, 2016; Neu et al., 2013; Moesch et al., 2016). Another study in Nile tilapia has shown that this species is capable of metabolizing dietary crude glycerol into lipids, proteins, and/or carbohydrates, as well as into CO_2_, to provide energy (Da Costa et al., 2017). A recent study demonstrated a positive effect of dietary glycerol for acting as a protein-sparing ingredient due to the decrease of amino acid catabolism in the carnivorous European seabass (*Dicentrarchus labrax*) (Rito et al., 2019).

Glycerol-3-phosphate (G3P) lies at the crossroad of glucose, lipid, and energy metabolism in vertebrates, actively participating in glycolysis and gluconeogenesis, lipid synthesis, and G3P electron transfer to mitochondria (Xue et al., 2017). Indeed, regarding glycerol metabolism in fish, it has been suggested that this compound is converted into glucose through the gluconeogenesis pathway, which can provide energy for cellular metabolism (Suarez and Mommsen, 1987; Brisson et al., 2001; Rito et al., 2019). Glycerol can be converted to G3P, which can enter the glycolytic pathway and the Krebs cycle to produce ATP (Pearson et al., 1990; Rito et al., 2019). In addition, glycerol is a precursor for the synthesis of triglycerides and phospholipids (Sheridan, 1988; Xue et al., 2017). Therefore, this simple sugar alcohol is pivotal for several metabolic pathways in fish as it is in other vertebrates.

The metabolic switching between feeding and fasting is central to life and involves tight regulation of metabolism including the one in the liver (Brisson et al., 2001). In the liver, aquaporin 9 (AQP9) facilitates the uptake of glycerol (Jelen et al., 2011; Lebeck, 2014). Glycerol kinase (Gk) catalyzes the initial phosphorylation of glycerol into G3P (Sriram et al., 2008), which can be used for gluconeogenesis after being reversibly oxidized by cytoplasmic G3P dehydrogenase (Gpd1) (Xue et al., 2017) into dihydroxyacetone phosphate (DHAP), a key intermediate of the glucose metabolism. Recently, Mugabo et al. (2016) identified a new pathway of glycerol metabolism in mammals with the identification of the G3P phosphatase (Pgp) that can directly hydrolyze G3P to glycerol. Indeed, in some fish species living in cold water (such as rainbow smelt *Osmerus mordax*), the conversion of G3P to glycerol by Pgp could be highly effective for their cryoprotection through sustained glycerol production (Ditlecadet and Driezdic, 2014; Driedzic, 2015). In cold-adapted Antarctic fish species, the use of glycerol as a precursor for gluconeogenesis was of low physiological importance (Magnoni et al., 2013) underlining how little is known about the regulation of glycerol metabolism, even under such extreme environments. Many of the enzymes involved in these processes are regulated by nutritional hormones (insulin and glucagon) such as Gk (Westergaard et al., 1998), Aqp9 (Lebeck, 2014), Pgp (Mugabo et al., 2016), and cytosolic Gpd1 (Patsouris et al., 2016). On a molecular level, only one study has been published in fish species (common carp *Cyprinus carpio*), which characterized the glycerol transporter gene, i.e., the one coding for the AQP9 (Dong et al., 2016). Until now, to our knowledge, no data are available concerning the molecular regulation of glycerol metabolism by nutrition in fish species.

Rainbow trout (*Oncorhynchus mykiss*) is a suitable experimental fish model, as this carnivorous species has an extensive and solid body of literature and is considered to be one of the most exploited species for freshwater aquaculture worldwide (Food Agric Organ, 2018). The provision of dietary energy in carnivorous fish derived from compounds other than from protein, such as glycerol, may reduce the use of dietary proteins as an energy source through catabolism (Panserat et al., 2009). Rainbow trout is one of the best models for fish nutrition and metabolism (Panserat et al., 2019). A preliminary study investigating the utilization of free glycerol in rainbow trout has been performed 34 years ago (Menton et al., 1986). Despite reporting some reduction in their feeding response, Menton et al. (1986) did not observe significant alteration in the final body weight and other zootechnical parameters nor in the biochemical composition of the carcass after 12 weeks of feeding. Unlike liver glycogen and plasma protein, blood glucose in rainbow trout provided with 6 and 12% glycerol-supplemented diets was more significant than that in control fish. Curiously, while the observed hyperglycemia at 3 h post-feeding returned to basal level at 18 h post-feeding in the fish supplemented with 6% glycerol, at 12% glycerol, fish maintained significantly higher blood glucose levels. This demonstrates an impairment of their capacity to clear the excess glucose at higher levels of dietary glycerol. Recent data about the effects of glycerol supplementation have been described in rainbow trout using a metabolomics approach (Palma et al., 2019). However, as explained before, the molecular regulation of glycerol metabolism by dietary glycerol for this fish species remains to be investigated. Our hypothesis is that glycerol metabolism is regulated at a molecular level by endogenous and/or exogenous nutrition. Thus, the objectives of the present study were (1) to characterize the glycerol metabolism-related genes using the rainbow trout genome database; (2) to analyze the ontogenesis of their gene expressions from the oocytes up to the first feeding (alevin stage); and to study the nutritional regulation of these genes (3) in the fasting–refeeding transition and the postprandial kinetic response after the last meal and (4) in continuous feeding with the inclusion of increasing levels of dietary glycerol (0, 2.5, and 5%).

## Materials and Methods

### Fish Experimental Design and Samplings

In order to test our hypothesis about the existence of nutritional regulation of glycerol metabolism in rainbow trout, the three following trials were used to study the ontogenesis, the postprandial kinetic, and the nutritional regulation for all the glycerol metabolism-related genes.

#### Fish Experimental Diet – Ontogenesis Sampling

Rainbow trout (*O. mykiss*) spawns were fertilized synchronously with neomale sperm and reared in separate tanks at 8°C at the INRA experimental facilities, Lées-Athas, France. Fish were sampled according to spawn origin before fertilization (oocyte) and then during development according to stages which are described by Vernier (1969): oocyte to stage 8, before embryonic genome activation (EGA); stage 10, EGA; stages 12 and 15, epiboly period; stage 22, primitive liver; stage 23, primitive hepatic portal vein; and stages 35–37, hatched embryo/endogenous feeding period. Endogenous feeding alevins (unfed fish) were sampled at 384°D (degree days, Vernier stage 31). Alevins at 616°D (Vernier stage 35) still exhibiting small yolk reserves were fed the first meal at 09:00 h and killed 3 h after the meal (corresponding to the postprandial period in alevins). The experimental feed, prepared in our facilities (Donzacq, France), contains crude protein (58.4% dry matter) and crude fat (17.5% dry matter), and gross energy is 21.7 kJ/g dry matter (for a detailed list of ingredients, see Marandel et al., 2016). Finally, 87-day-old alevins (696°D at 8°C), characterized by complete resorption of the yolk sac (Vernier stage 37) and maintained on a continuous feeding regime of several meals a day, were killed 3 h following the final feeding as described above. Care was taken to maintain the same daily time frame before samplings to avoid potential circadian effects, known to affect metabolism in rainbow trout (Bolliet et al., 2000). Fish were therefore consistently sampled at 12:00 h for each ontogenetic stage investigated in this study, as previously described (Mennigen et al., 2013). Embryos were directly snap-frozen, whereas alevins were killed by terminal anesthetization by bathing in benzocaine before pooling and storage in liquid nitrogen. We used *N* = 3 pools of 30 embryos, each pool coming from one female from stages O to 23 and then *N* = 3 alevins per spawn (in total, *N* = 9) from stages 31 to 37. The samples were stored at −80°C until analyzed.

#### Fish Experimental Diet – Postprandial Sampling

Prior to the experiment, rainbow trout (*O. mykiss*) fish had initially been reared in our experimental facilities (INRA, Donzacq, France) at 18°C and fed a commercial diet (Skretting, France; crude protein: 49.8% dry matter, crude fat: 13.8% dry matter, gross energy: 22 kJ/g dry matter). Immediately prior to the experiment, fish were fasted for 48 h, to allow for basal metabolite plasma concentrations to be reached. In trout, these basal metabolite concentrations are typically reached more slowly compared to endothermic mammals due to slower intestinal transit and gastric emptying. Following the fast, fish were fed once *ad libitum* with the commercial diet. Six trout were sampled for each time point, starting with 48 h fasted fish at 0 h and following feeding at 2, 4, 8, 12, 16, and 24 h, as previously described (Mennigen et al., 2012). Immediately following a terminal anesthetization, the liver was dissected and frozen in liquid nitrogen prior to storage at −80°C until analyzed. Gut content of the sampled animals was checked to verify that the fish had effectively consumed the diet.

#### Fish Experimental Diet – Nutritional Sampling

Juvenile rainbow trout (*O. mykiss*) were reared in tanks at the Experimental Research Station (Vila Real, Portugal) of the University of Trás-os-Montes e Alto Douro (UTAD) facilities in freshwater according to standard juvenile rearing protocols. At the beginning of the experiment, fish were fasted for 24 h and were then divided into triplicate groups of 25 fishes (225 fish in total, BW 20.22 ± 0.06 g) randomly distributed among nine fiberglass tanks in an open circulation water system (14.6 ± 0.1°C). Three experimental diets [SPAROS LDA, Olhão, Portugal; isoproteic (49% dry matter) and isoenergetic (gross energy: 21 kJ/g dry matter)] were formulated to give a contrast in the glycerol content by supplementing a basal diet (control) with this compound at 2.5 or 5% w/w; Magnoni et al., unpublished data. Glycerol used in the formulation of the diet (refined glycerine) was obtained from rapeseed (Belgosuc, 050008, Beernem, Belgium). The incorporation of cellulose at different levels in the formulation was used to create the dietary contrast for the addition of glycerol. Therefore, cellulose was used as an indigestible filler (Hilton, 1982), which does not affect the utilization of dietary energy or protein in rainbow trout (Bromley and Adkins, 1984; Wiesmann and Pfeffer, 1986). The fish were hand-fed *ad libitum* 6 days/week and twice per day (9:00 and 17:00 h). Fish survival was 99, 100, and 99% for groups assigned to control, 2.5, and 5% glycerol diets, respectively. All the fish were individually weighed at the end of the feeding trial (60 days), and no differences were detected on the final BW between groups (Magnoni et al., unpublished data). Two to three fish per tank were sampled 6 h after the morning feeding (*n* = 8) and 24 h after the morning feeding (*n* = 8). Immediately following a terminal anesthetization, the liver was dissected, weighed, and immediately frozen in liquid nitrogen and stored at −80°C until analyzed.

### *In silico* Analysis

**FIGURE 1:**
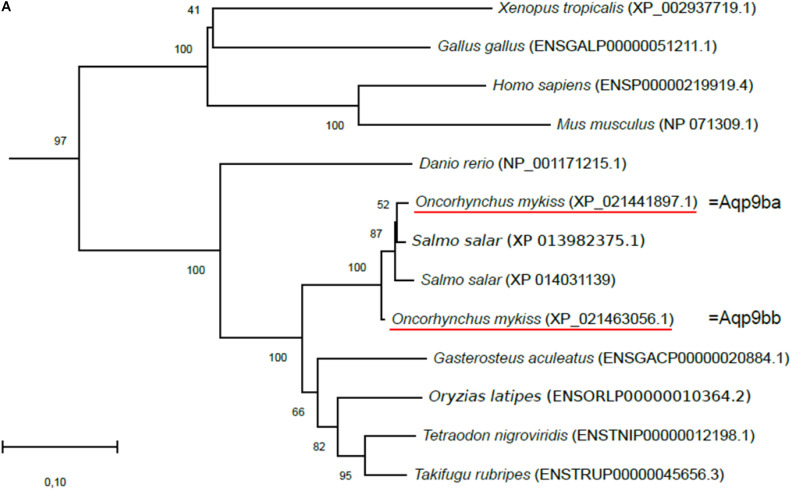

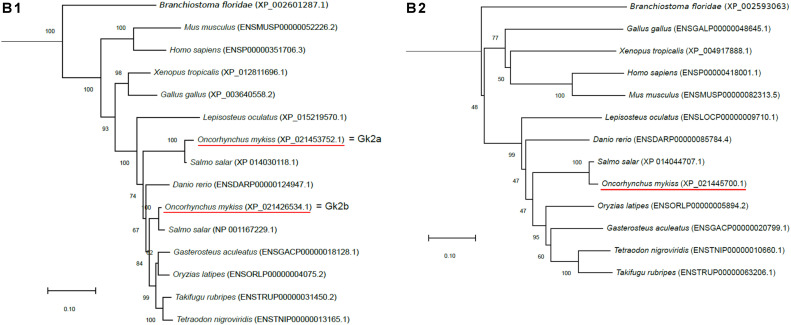

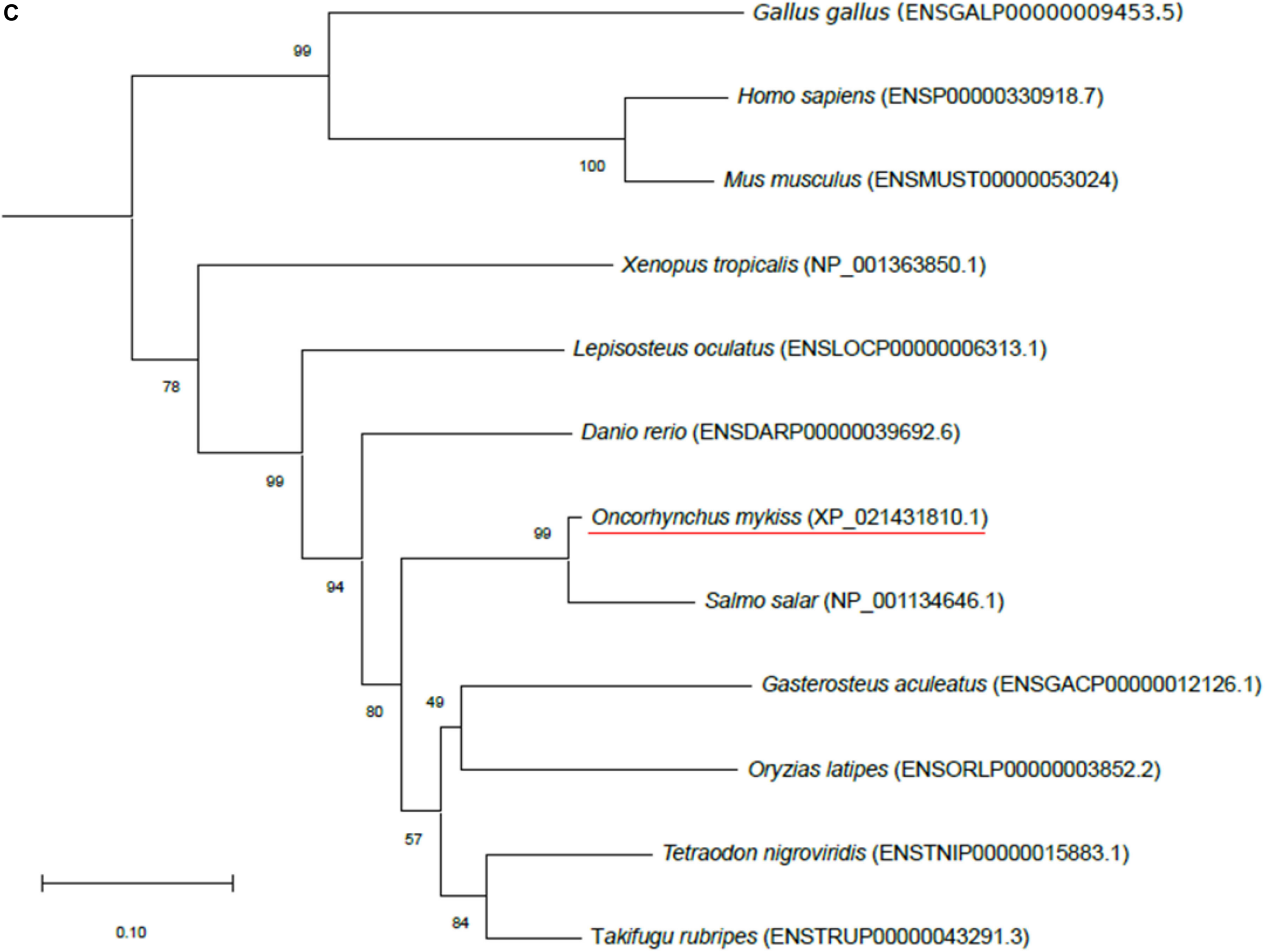

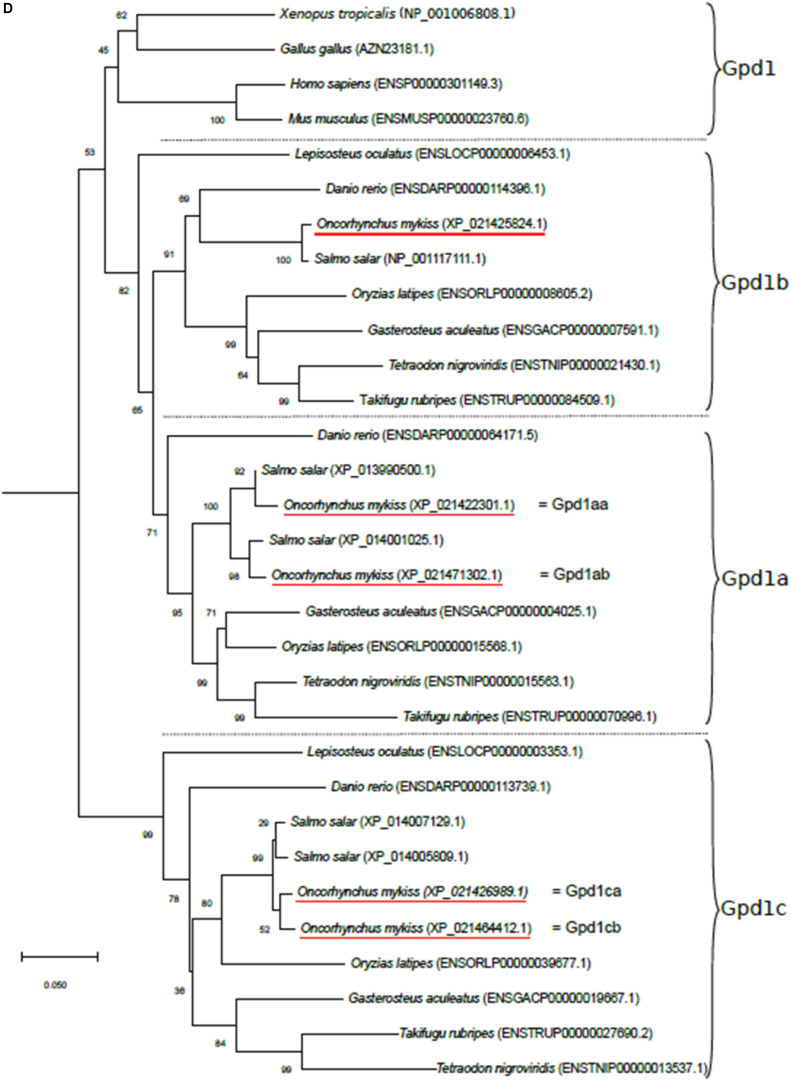
Phylogenetic analysis of **(A)** aquaporin 9b (glycerol transport, *aqp9b*), **(B)** glycerol kinases (*gk2* and *gk5*), **(C)** glycerol-3-phosphate phosphatase (*pgp*), and **(D)** glycerol-3-phosphate dehydrogenases 1 (*gpd1a*, *gpd1b*, and *gpd1c*). The full-length deduced amino acid sequence was aligned using MUSCLE software (Edgar, 2004). Phylogenetic analysis was performed using the Molecular Evolutionary Genetics Analysis (MEGA) v6 software (Tamura et al., 2013). Phylogenetic trees were built using the neighbor-joining (NJ) method. The reliability of the interfered trees was estimated using the bootstrap method with 1,000 replications. NCBI or Ensembl protein accession numbers are given in parentheses. **(A)** Aqp9b tree was rooted with Aqp7 protein sequences, **(B1)** Gk2 tree was rooted with Gk5 protein sequences, **(B2)** Gk5 was tree rooted with Gk2 protein sequences, **(C)** Pgp tree was rooted with the *Branchiostoma floridae* (XP_002608555) protein sequence, and **(D)** Gpd tree was rooted with the *B. floridae* (XP_002603247.1) protein sequence.

Rainbow trout *aquaporin 9b* (*aqp9b*), *glycerol kinase* (*gk*), *G3P phosphatase* (*pgp*), and *G3P dehydrogenase 1* (*gpd1*) genes were extracted from the *O. mykiss* genome assembly NCBI database (GCF_002163495.1, June 2017) using the zebrafish (*Danio rerio*) orthologous sequence as a query sequence in the BLAST tool (rainbow trout accession numbers are given in Table 1). Phylogenetic analysis (Figure 1) was performed using the Molecular Evolutionary Genetics Analysis (MEGA) package v6 software (Tamura et al., 2013). To do so, sequences from species other than zebrafish and trout were collected from the Ensembl Genome database (Ensembl release 99, January 2020)^1^ or NCBI. Phylogenetic trees, based on deduced full-length amino acid sequences, were produced using the neighbor-joining (NJ) method and confirmed by the maximum likelihood method (data not shown). Reliabilities of the interfered trees were estimated using the bootstrap method with 1,000 replications.

**TABLE 1:** List of the glycerol metabolism-related genes (*glycerol kinase* – *gk*; *aquaporin 9b – aqp9b*; *glycerol-3-phosphate phosphatase* – *pgp*; and *glycerol-3-phosphate dehydrogenase 1* – *gpd1*) and their associated primers for quantitative RT-PCR in rainbow trout.

**Genes**	**5′**/**3′ Forward primer**	**5′**/**3′ Reverse primer**	**Accession numbers**
**Reference gene**			
Luciferase control	CATTCTTCGCCAAAAGCACTCTG	AGCCCATATCCTTGTCGTATCCC	
*EF1*α	TCCTCTTGGTCGTTTCGCTG	ACCCGAGGGACATCCTGTG	AF498320
**Glycerol metabolism**			
*aqp9ba*	TGCTGGATCTTGTGCTGTCT	CAATGCACAGTGGCTCCAT	XM_021586222.1
*pgp*	CTGAGGTGAAGGCTGTGGTT	GGCCACACAGTCGTACATGA	XM_021576135.1
*gk2a*	TGGGATAAAGCAGAGTTTCCC	CCGTCTCTTTGTCCCAAACCA	XM_021598077.1
*gk5*	AAGCCCAATGACTGCCTCTA	TGCATCCTCCTTCGTCCTAT	XM_021590025
*gpd1aa*	CAACTCCCAAGAAAGTCTGCA	GATGTCTGTCAGCTTGCGAC	XM_021566626.1
*gpd1b*	CAAGAACAAAGAGTGGGGCC	ATCATCTCCATCAGCCCCAG	XM_021570149.1
*gpd1c*	TTGGATCTGGAAACTGGGGC	AGTATGCTGGCCCCTTTCAC	XM_021571314.1 and XM_021608737.1

*gpd1c corresponds to the global amplification of the arbitrarily named gpd1ca and gpd1cb genes.*

### mRNA Level Analysis

The relative gene mRNA level was determined by quantitative real-time RT-PCR. Samples were homogenized using Precellys^®^ 24 (Bertin Technologies, Montigny-le-Bretonneux, France) in tubes containing TRIzol reagent (Invitrogen, Carlsbad, CA, United States). For ontogenesis experiment only, luciferase control RNA (Promega, Charbonnières, France), 10 pg per 1.9 mg of embryo/alevin or oocyte, was added to each sample to allow for data normalization during early development as previously described (Desvignes et al., 2011; Marandel et al., 2012). Total RNA was then extracted according to the manufacturer’s instructions. The total RNA concentrations were determined using the spectrophotometer NanoDrop 2000, whereas the total RNA qualities were determined after migration on a 1% agarose gel electrophoresis. Total RNA (1 μg) was used for cDNA synthesis. For the postprandial and nutritional experiment, luciferase control RNA (Promega) was added during the reverse-transcription step (1 pg/μg of RNA). The SuperScript III RNase H-Reverse transcriptase kit (Invitrogen, Carlsbad, California, United States) was used with random primers (Promega) to synthesize cDNA.

The primer sequences used in real-time RT-PCR assays for glycerol metabolism gene analysis were designed and are listed in Table 1. We tried to amplify all the genes (see ahead, *In silico* Analysis, in “Results” section), but we only succeeded for genes described in Table 1. They were validated on a pool of cDNA, and amplified products were sequenced systematically. Sequencing the qPCR products amplified by *gpd1c*-related primers, we highlighted some polymorphism showing that the two genes *gpd1ca* (XM_021571314.1) and *gpd1cb* (XM_021608737.1) were amplified by the set of primers. These two nucleotidic sequences shared 94.8% identity, thus highly conserved. Moreover, our phylogenetic tree analysis (Figure 1D) strongly suggested that *gpd1ca* and *gpd1cb* were orthologous genes emerging from the recent salmonid-specific whole-genome duplication and that they potentially had not diverged. As shown in previous studies, trout ohnolog genes were mostly regulated at the transcription level in the same way (Marandel et al., 2015). We thus decided to use the set of primers that amplify both *gpd1ca* and *gpd1cb* genes.

For real-time RT-PCR assays, the Roche LightCycler 480 system was used (Roche Diagnostics, Neuilly-sur-Seine, France). The assays were performed using a reaction mix of 6 μl per sample, each of which contained 2 μl of diluted cDNA template (1:76 for the postprandial and nutritional experiment; 1:25 for the ontogenesis experiment), 0.24 μl of each primer (10 μmol/L), and 3 μl of LightCycler 480 SYBR^®^ Green I Master mix (Roche Diagnostics). The PCR protocol was initiated at 95°C for 10 min for initial denaturation of the cDNA and hot-start enzyme activation and continued with 45 cycles of a three-step amplification program (15 s at 95°C followed by 10 s 60°C, for primer hybridization, and 15 s at 72°C to extend DNA). Melting curves were systematically monitored (temperature gradient at 0.11°C/s from 65 to 97°C; five acquisitions per degree Celsius) at the end of the last amplification cycle to confirm the specificity of the amplification reaction. Each PCR run included replicate samples (duplicate of reverse transcription and duplicate of PCR amplification) and negative controls (reverse transcriptase-free samples and RNA-free samples).

### Relative Quantification of mRNA Levels

For the analysis of mRNA levels, relative quantification of target gene expression was performed using the Roche Applied Science *E*-Method. For ontogenesis and postprandial experiments, the target gene was normalized with exogenous luciferase transcript abundance (Promega). For nutritional experiments, the target gene was normalized with *elongation factor 1*α (*EF1*α) measured RNA. As the relative expression of luciferase control and *EF1*α did not significantly change over the sampling process (data not shown), in all cases, PCR efficiency was measured from the slope of a standard curve using serial dilutions of cDNA; PCR efficiency values ranged between 1.8 and 2.0.

### Statistical Analysis

Molecular data about ontogenesis and postprandial experiments were analyzed by one-way analysis of variance (ANOVA). When significant differences were found among the groups (*P* < 0.05), Tukey’s procedure was used to rank the groups. Molecular and plasma parameters of the dietary glycerol experiment were analyzed by two-way ANOVA. In all these analyses, data sets were tested for normality (Shapiro–Wilk) and equal variance (Levene’s test). Values are reported as means ± SD. Data analyses were carried out using R software (version 3.4.4).

## Results

### *In silico* Analysis of Rainbow Trout *aqp9b*, *gk*, *pgp*, and *gpd1* Genes

By analyzing the assembly of the rainbow trout genome on NCBI, we identified for the first time several genes sharing high sequence homology with zebrafish glycerol pathway-related genes: two sequences were related to *aqp9b*, three to *gk*, one to *pgp*, and five to *gpd1*. Phylogenetic analyses, using full-length protein sequences, were performed to confirm the identity of trout sequences (Figure 1).

#### Aquaporin 9b

Two sequences were identified in the trout genome sharing 70.9% (XP_021441897.1) and 71.6% (XP_021463056.1) identity with the zebrafish Aqp9b (ENSDARP00000069995.5) in terms of amino acid sequences. These two sequences were highly conserved with 97% identity in common. Our phylogenetic tree showed that the two trout sequences grouped with the vertebrate Aqp9b orthologous sequences and were close to the two Atlantic salmon (*Salmo salar*) *aqp9b* genes (Figure 1A). Together these results confirmed that the two sequences identified in the rainbow trout genome were *aqp9*-related ones. We arbitrarily annotated the two rainbow trout aquaporin 9 genes *aqp9ba* (NCBI ID: 110506537) and *aqp9bb* (NCBI ID: 110526415). The two sequences identified in salmonids (vs. only one in other teleost fish) suggested that this gene duplicated before or around the salmonid radiation following the salmonid whole-genome duplication (SaGD, Berthelot et al., 2014).

#### Glycerol Kinase

Two zebrafish glycerol kinase-related genes were identified in the Ensembl database as *gk2* (Ensembl accession no. ENSDARG00000053456) and *gk5* (Ensembl accession no. ENSDARG00000062864). By blasting them against the NCBI rainbow trout assembly analysis, we found two sequences related to *gk2* and one related to *gk5* (NCBI ID: 110509068). Gk2-related sequences were highly conserved (86% identity) and shared 86 and 92% identity with zebrafish Gk2. Our phylogenetic tree showed that the two trout sequences grouped with vertebrate Gk2 orthologous and were close to the two Atlantic salmon (*S. salar*) *gk2* genes (Figure 1B). As for Aqp9, this configuration was in favor of a salmonid-specific duplication occurring after SaGD. We arbitrarily called them *gk2a* (NCBI ID: 110520656) and *gk2b* (NCBI ID: 110495542).

For Gk5, the trout sequence shared 76.7% homology with the zebrafish Gk5. Again, its place in the phylogenetic tree (Figure 1B) confirmed its Gk5 identity in trout.

#### G3P Phosphatase

Only one sequence was identified in the rainbow trout genome using NCBI BLAST (XP_021431810.1), which shared 79.7% identity with zebrafish Pgp (ENSDARP00000039692.6). This sequence grouped with Pgp vertebrate orthologs in our phylogenetic analysis (Figure 1C) confirming its identity as a Pgp protein in trout.

#### G3P Dehydrogenase 1

Three zebrafish G3P dehydrogenase 1 genes were identified in the Ensembl database: *gpd1a* (Ensembl accession no. ENSDARG00000043701), *gpd1b* (Ensembl accession no. ENSDARG00000043180), and *gpd1c* (Ensembl accession no. ENSDARG00000036942). Blasting them against the NCBI rainbow trout assembly, we identified two sequences related to *gpd1a*, both sharing 83% identity with the zebrafish sequence; one related to *gpd1b* (NCBI ID: 110494817), sharing 85% identity with the zebrafish sequence; and two related to *gpd1c*, sharing 84.8 and 84% identity with the zebrafish one. Our phylogenetic analysis (Figure 1D) showed that the trout sequences grouped with the corresponding orthologs in other vertebrates. Together, our results confirmed the identity of the identified sequences in trout. Finally, the two trout *gpd1a*-related sequences were arbitrarily named *gpd1aa* (NCBI ID: 110492370) and *gpd1ab* (NCBI ID: 110532036), and the two *gpd1c*-related sequences were arbitrarily named *gpd1ca* (NCBI ID: 110495844) and *gpd1cb* (NCBI ID: 110527462).

Based on these molecular findings and because we did not succeed to amplify some of the mRNAs (for *aqp9bb*, *gk2b*, and *gpd1ab* duplicated genes), transcript accession numbers corresponding to genes described above are listed in Table 1.

### Ontogenesis of Glycerol Gene Expression in Rainbow Trout

Real-time PCR was performed to determine the stage-specific expression of rainbow trout glycerol-related genes during embryogenesis and in alevins. Stages were defined based on the Vernier development table (Vernier, 1969) and chosen to target developmental periods of interest for metabolism. In Table 2A, this analysis showed that mRNA levels of *pgp*, *gk5*, *gpd1c*, and *gpd1aa* glycerol-related genes increase significantly at stage 22 (primitive liver) up to stage 30 (hatched embryo). The significant increase of *aqp9b* and *gpd1b* genes appears later at stage 30. The *gk2a* gene has a very different profile of expression during ontogenesis; indeed, the mRNA level of the *gk2a* gene decreases significantly at stage 10 (embryonic genome activation) compared to the oocyte stage. During the nutritional transition (Table 2B), we observed an increase of mRNA levels for *gk2a*, *gk5*, *gpd1b*, and *gpd1c* genes at stage 35 (stage of mixed endogenous–exogenous feeding). Compared to stage 35, there is a significant decrease of expression of the glycerol genes (except the *gk2a*) when rainbow trout are fed with exogenous diet (58% protein and 17.5% lipids) (stage 37).

**TABLE 2:** Ontogenesis of glycerol metabolism-related genes in the whole body of rainbow trout before the first feeding stage.

**Target genes**	**Developmental Stages**							
	**Oocyte**	**5**	**6**	**7**	**8**	**10**	**12**	**15**
*aqp9ba*	0.110.00^a^	0.010.01^a^	0.010.00^a^	0.000.00^a^	0.010.01^a^	0.000.00^a^	0.000.00^a^	0.000.00^a^
*pgp*	0.010.02^a^	0.010.01^a^	0.010.01^a^	0.000.00^a^	0.050.04^a^	0.060.09^a,b^	0.290.29^a,b^	0.390.11^*b*^
*gk2a*	3.331.62^a^	2.281.48^a,b^	1.830.86^a,b^	2.731.73^a,b^	2.681.56^a,b^	1.380.50^a,b^	0.280.19^*b*^	0.180.02^*b*^
*gk5*	0.040.05^a^	0.050.05^a,b^	0.040.07^a,b^	0.030.04^a^	0.050.08^a,b^	0.020.03^a^	0.040.03^a^	0.050.02^a,b^
*gpd1aa*	0.040.02^a^	0.020.01^a^	0.010.00^a^	0.020.01^a^	0.030.02^a^	0.010.00^a^	0.030.02^a^	0.040.01^a^
*gpd1b*	0.030.02^a^	0.030.02^a^	0.020.01^a^	0.030.03^a^	0.040.03^a^	0.030.02^a^	0.010.00^a^	0.010.01^a^
*gpd1c*	0.050.04^a^	0.050.02^a^	0.040.04^a^	0.060.04^a^	0.050.04^a^	0.050.03^a^	0.030.01^a^	0.070.02^a^

	**22**	**23**	**31**	***p*-value**				

	0.010.01^a^	0.020.01^a^	0.220.03^*b*^	6.30E-16				
	1.630.17^*c*^	1.750.05^*c*^	1.540.10^*c*^	< 2E-16				
	0.680.12^a,b^	0.570.08^a,b^	0.540.19^a,b^	0.005				
	0.430.17^*b*,*c*^	0.470.17^*c*^	0.630.34^*c*^	1.45E-5				
	2.370.34^*c*^	3.200.17^*d*^	1.250.19^*b*^	< 2E-16				
	0.070.02^a^	0.090.03^a^	1.090.15^*b*^	< 2E-16				
	0.800.12^*b*^	0.830.04^*b*^	0.770.20^*b*^	8.44E-14				

*The stages are those described by Vernier (1969). Description of the stages: oocyte to stage 8, before embryonic genome activation (EGA); stage 10, EGA; stages 12 and 15, epiboly period; stage 22, primitive liver; and stage 23, primitive hepatic portal vein. gpd1c corresponds to the global amplification of the arbitrarily named gpd1ca and gpd1cb genes. Different letters are shown when the mRNA abundances are significantly different. Values are reported as means ± SD.*

**TABLE 2:** Ontogenesis of glycerol metabolism-related genes in the whole body of rainbow trout during the nutritional transition (first feeding stage).

**Target genes**	**Developmental stages (first feeding stage)**			***p*-value**
	**31**	**35**	**37**	
*aqp9ba*	0.22 ± 0.06^a^	2.04 ± 1.36^b^	1.05 ± 0.36^c^	1.02E-7
*pgp*	1.56 ± 0.34^a^	1.80 ± 1.38^a^	0.79 ± 0.21^b^	1.48E-3
*gk2a*	0.55 ± 0.22^a^	1.48 ± 1.05^b^	1.17 ± 0.51^b^	6.83E-4
*gk5*	0.64 ± 0.42^a^	1.64 ± 1.51^b^	0.75 ± 0.36^a^	3.99E-3
*gpd1aa*	1.26 ± 0.34^a,b^	1.72 ± 1.14^b^	0.70 ± 0.14^a^	2.67E-4
*gpd1b*	1.11 ± 0.34^a^	2.04 ± 1.31^b^	0.78 ± 0.20^a^	4.64E-5
*gpd1c*	0.79 ± 0.29^a^	1.86 ± 1.64^b^	0.96 ± 0.23^a^	0.0033

*The stages are those described by Vernier (1969). Description of the stages: stages 31–35–37, hatched embryo/endogenous feeding period. gpd1c corresponds to the global amplification of the arbitrarily named gpd1ca and gpd1cb genes. Different letters are shown when the mRNA abundances are significantly different. Values are reported as means ± SD.*

### Postprandial Regulation of Glycerol Metabolism-Related Gene Expression in Rainbow Trout Liver

Table 3 shows the postprandial kinetic of the glycerol metabolism-related gene expression after feeding (commercial diet: 42% of proteins and 22% of lipids). Postprandial times had a significant effect on the expressions of *pgp*, *gk2a*, *gpd1aa*, and *gpd1b* genes but no effect on *aqp9ba*, *gk5*, and *gpd1c* genes (Table 3). Indeed, hepatic expression of glycerol metabolism-related genes (*pgp*, *gk2a*, and *gpd1aa*) revealed significant postprandial increases in expression after refeeding (2 h after the last meal for *gk2a* and > 8 h after the last meal for the other genes), while *gpd1b* decreased 4 h after the last meal.

**TABLE 3:** Postprandial regulation of glycerol metabolism gene expression in rainbow trout liver. 0 h: fasted fish 48 h. 2, 4, 8, 12, 16, and 24 h after the last meal.

**Target genes**	**0 h**	**2 h**	**4 h**	**8 h**	**12 h**	**16 h**	**24 h**	***p*-value**
*aqp9ba*	1.150.68	0.980.43	0.680.35	1.040.16	0.820.37	0.830.45	0.970.32	0.543
*pgp*	0.170.05^a^	0.370.06^a,b^	0.800.26^a,c^	1.160.19^b,c^	1.240.62^b,c^	1.540.77^c^	0.930.57^a,c^	5.83E-05
*gk2a*	0.450.18^a^	1.120.36^b,c^	0.720.18^a,c^	1.340.48^c^	1.000.26^a,c^	1.100.65^a,c^	0.720.24^a,b^	0.003
*gk5*	1.290.13	1.162.71	0.050.04	1.601.25	0.941.28	0.491.12	1.151.18	0.317
*gpd1aa*	0.390.21^a^	0.470.17^a,b^	0.460.20^a^	0.890.19^b^	0.750.21^a,b^	0.820.28^a,b^	0.470.27^a,b^	6.00E-04
*gpd1b*	0.760.24^a^	0.630.18^a,b^	0.420.15^b^	0.690.17^a,b^	0.480.11^b^	0.690.25^a,b^	0.640.15^a,b^	0.031
*gpd1c*	0.850.22	0.840.26	0.760.31	1.130.30	0.960.33	1.130.30	1.220.41	0.101

*gpd1c corresponds to the global amplification of the arbitrarily named gpd1ca and gpd1cb genes. Different letters are shown when the mRNA abundances are significantly different. Values are reported as means ± SD.*

### Regulation of Glycerol Metabolism-Related Gene Expression by Dietary Glycerol in Rainbow Trout Liver

The last part of the experiments was dedicated to studying the regulation of the glycerol metabolism-related gene expression by dietary glycerol. We fed rainbow trout for 8 weeks with 0, 2.5, and 5% of glycerol. We analyzed the gene expression 6 and 24 h after the last meal. As reflected by the plasma analysis of glycerol, glucose, and triglycerides, there was an expected increase of plasma glycerol in fish fed 5% of glycerol but only 6 h after the last meal (Table 4); higher levels of nutrient absorption 6 h after the last meal are confirmed by the increase of glycemia 6 h after the last meal vs. 24 h after the last meal. Regarding the effects of dietary glycerol on the mRNA levels for glycerol metabolism-related genes, there were no significant effects on their levels (Table 5). Only an effect linked to the postprandial timing was observed for the *pgp* gene which was more highly expressed at 6 h compared to 24 h after the last meal.

**TABLE 4:** Plasma metabolites in rainbow trout fed with different levels of glycerol (0, 2.5, and 5%) for 60 days.

**Metabolites**	**6 h**			**24 h**			***p*-value**		
	**0%**	**2.5%**	**5%**	**0%**	**2.5%**	**5%**	**Diet**	**Time**	**Diet × Time**
Glucose (g/L)	0.78 ± 0.10	0.83 ± 0.13	0.74 ± 0.18	0.73 ± 0.09	0.68 ± 0.08	0.67 ± 0.08	0.419	0.011	0.436
Triglyceride (g/L)	3.25 ± 0.86	3.44 ± 1.73	5.09 ± 1.68	2.97 ± 1.37	3.13 ± 1.72	3.47 ± 1.32	0.065	0.091	0.349
Glycerol (mM)	0.05 ± 0.00	0.06 ± 0.01	0.08 ± 0.05	0.04 ± 0.01	0.04 ± 0.01	0.04 ± 0.00	0.131	9.27E-08	1.46E-03

*Values are reported as means ± SD.*

**TABLE 5:** Regulation of glycerol metabolism gene expression in rainbow trout liver by dietary glycerol for 60 days at 6 and 24 h after the last meal.

**Target genes**	**6 h**			**24 h**			***p*-value**		
	**0%**	**2.5%**	**5%**	**0%**	**2.5%**	**5%**	**Diet**	**Time**	**Diet × Time**
*aqp9ba*	0.91 ± 0.37	0.94 ± 0.49	1.17 ± 0.72	0.94 ± 0.59	0.99 ± 0.63	1.27 ± 0.75	0.377	0.751	0.987
*pgp*	1.53 ± 0.71	1.68 ± 0.44	1.61 ± 0.43	0.47 ± 0.21	0.38 ± 0.15	0.44 ± 0.15	0.983	1.17E-11	0.657
*gk2a*	1.06 ± 0.49	0.95 ± 0.47	0.90 ± 0.39	1.21 ± 0.23	0.93 ± 0.47	1.04 ± 0.65	0.499	0.530	0.848
*gk5*	1.56 ± 1.33	0.87 ± 0.26	0.86 ± 0.68	1.04 ± 0.77	1.31 ± 0.55	0.94 ± 0.53	0.366	0.999	0.220
*gpd1aa*	0.83 ± 0.48	1.09 ± 0.37	1.2 ± 0.46	0.91 ± 0.21	0.77 ± 0.30	0.83 ± 0.29	0.587	0.076	0.206
*gpd1b*	0.89 ± 0.40	1.10 ± 0.45	1.03 ± 0.41	0.94 ± 0.30	0.84 ± 0.26	1.20 ± 0.23	0.326	0.892	0.255
*gpd1c*	0.96 ± 0.19	1.14 ± 0.33	1.23 ± 0.51	0.93 ± 0.13	0.94 ± 0.28	0.97 ± 0.14	0.372	0.082	0.574

*gpd1c corresponds to the global amplification of the arbitrarily named gpd1ca and gpd1cb genes. Values are reported as means ± SD.*

## Discussion

Glycerol metabolism is at the interface of lipid and glucose metabolism (Mugabo et al., 2016; Xue et al., 2017; Possick et al., 2017). Up to now, data about genes of glycerol metabolism in rainbow trout (and in other fish species) are scarce; in particular, no studies have been published concerning its molecular regulation. The recent sequencing of the rainbow trout genome should provide new tools to investigate glycerol metabolism in trout, taking into account that this genome has recently suffered a whole duplication event (Berthelot et al., 2014). The present study characterizes the rainbow trout genes coding aquaporin 9 (*aqp9b* and *aqp9b* genes), glycerol kinase (*gk2a*/*gk2b* and *gk5* genes), G3P phosphatase (*pgp* gene), and cytosolic G3P dehydrogenase (*gpd1* genes, i.e., *gpd1aa*, *gpd1ab*, *gpd1b*, *gpd1ca*, and *gpd1cb* genes) using the genomic sequence of the rainbow trout. Therefore, the present investigation aimed to determine, for the first time in a teleost species, the regulation of *gk2a*, *pgp*, *gk5*, *gpd1aa*, *gpd1b*, and *gpd1c* gene expressions during developmental stages and with the nutritional environment.

### mRNA Levels of Glycerol Metabolism-Related Genes Increase During Ontogenesis From the Setup of the Primitive Liver in Rainbow Trout

Following the characterization of the different genes of the glycerol pathway, the objective of the present paper was to describe the regulation of expressions of these genes during ontogenesis. We observed higher expression of these genes (*pgp*, *gk2a*, *gk5*, *gpd1aa*, *gpd1b*, and *gpd1c*) mainly from stages 22 and 23, corresponding to the setup of the primitive liver and the primitive hepatic portal vein, respectively (Vernier, 1969). These data are not surprising because glycerol metabolism-related genes are known to be mainly expressed in the liver of mammals (Possick et al., 2017). For unclear reasons (no need of glycerol transport at this stage?), the *aqp9b* and *gpd1b* gene expressions appear later at stage 30 just before the first feeding stage. On the other hand, the *gk2a* gene has a particular profile of expression during ontogenesis: indeed, higher levels of *gk2a* mRNAs were observed at the very first stages of the development (i.e., from oocyte stage up to stage 10—blastula), suggesting that this mRNA was maternally inherited. The *gk2a* gene may play an important role during the early cleavage period in rainbow trout. Indeed, an important role of *gk2*a could be to sustain embryo with G3P for its early synthesis of complex lipids (Terner et al., 1968).

### Hepatic Glycerol Metabolism-Related Gene Expressions Are Upregulated After Feeding in Trout Alevins and Juveniles

The first feeding stage is a stage of high molecular plasticity in fish metabolism (Mennigen et al., 2012; Marandel et al., 2016). Indeed, the increase of mRNA levels for all the glycerol metabolism-related genes (*aqp9ba*, *gk2a*, *gk*5, *pgp*, *gpd1aa*, *gpd1b*, and *gpd1c*) was observed during the endogenous–exogenous feeding transition (stage 35). Moreover, by contrast, when the yolk reserves were totally consumed (stage 37), i.e., when the fish were fed only with inert diets, there is a decrease in the levels of many of the studied mRNAs. Just before the end of the resorption of the yolk reserves (stage 35), the majority of the remaining nutrients are lipids and especially triacylglycerol (Kamler, 2008). Yolk triglycerides would be the main endogenous stores at stage 35, with the preferential utilization by the alevins of fatty acids and the possible use of 3-carbon backbone of glycerol for glucose synthesis through gluconeogenesis (Vernier and Sire, 1976) as described in mammals and fish (Rito et al., 2019). Despite several methodological caveats, the use of glycerol with carbon isotopes, either delivered in labeled pellets (Da Costa et al., 2017) or delivered in incubation medium for hepatocytes (Lech, 1970) or IP injected (Savina and Wojtczak, 1977; Rito et al., 2019), assisted greatly in confirming that fish species are able to catabolize it as well as retain and incorporate it into carbohydrate, namely, glucose via gluconeogenesis. This use of glycerol from endogenous feeding could explain the increase of the glycerol metabolism-related gene expressions at this stage. Later (i.e., stage 37 here), when the yolk is fully consumed and its energetic content depleted, their nutrition is from an inert commercial feed consisting of 58% of protein and 17% of lipids (Marandel et al., 2016). Contrary to stage 35, the alevins of this carnivorous species can use, and even prioritize, the catabolism of amino acids as the main energy source, being a possible explanation for the lower levels of glycerol metabolism-related gene expression.

It was also very important to know about the postprandial regulation of glycerol metabolism-related gene expression in the liver as it may reflect glycerol absorption, transport, sensing, and processing mechanisms. Indeed, it is well known that some metabolic genes can be strongly regulated in the liver after the last meal as shown for example with some hepatic genes coding key enzymes of intermediary metabolism (Mennigen et al., 2012). Here, in rainbow trout juveniles fed with a commercial diet (44% proteins and 22% lipids), the significant postprandial change of gene expressions is only observed for four hepatic genes of the glycerol metabolic pathway: *pgp*, *gk2a*, *gpd1c*, and *gpd1aa*. The postprandial increase of mRNA abundances for *pgp*, *gk2a*, and *gpd1aa* is observed between 2 and 16 h after the last meal. This kinetic of postprandial regulation is similar to the one observed previously for some nutritionally dependent gene expressions (for example, for glycolytic and gluconeogenic genes) (Mennigen et al., 2012). These data strongly suggest that glycerol metabolism could be dependent on the nutritional status of fish.

### Hepatic Glycerol Metabolism-Related Gene Expressions Are Poorly Controlled by Different Levels of Dietary Glycerol

Even though the glycerol metabolism is dependent on the nutritional status (see before), when we fed the fish with different levels of dietary glycerol (0, 2.5, and 5%), we did not observe any significant effects on the hepatic glycerol metabolism-related gene expression (i.e., for the *gk2a*, *gk5*, *pgp*, *gpd1aa*, *gpd1b*, and *gpd1c* genes). Even though the significant increase 6 h after the last meal of the plasma glycerol with dietary glycerol levels proves that glycerol was absorbed at the intestine level and so, in theory, available for utilization in the liver, we must note here that the values of the plasma glycerol are low (max 0.08 mM in trout fed 5% glycerol) if we compared these data with European seabass fed the same diets (up to 6 mM of plasma glycerol; unpublished data). It is hypothesized that the levels of plasma glycerol were not sufficiently high in rainbow trout fed different levels of dietary glycerol to be able to modify the levels of hepatic gene expression from glycerol metabolism. An NMR-based metabolomic analysis using the remaining fish from the nutritional sampling here presented seemed to corroborate these observations. It revealed that the hepatic metabolome in trout was also poorly modified by these levels of dietary glycerol (Palma et al., 2019), which articulates well with the present molecular data. Despite this apparent weak effect on hepatic metabolism, it is noteworthy that the triacylglycerol turnover rate in rainbow trout was relatively high (Magnoni et al., 2008). Instead of being metabolized, glycerol can be perceived as excess carbohydrate and be diverted to support triacylglycerol synthesis. This seems to be the case in trout as in other carnivorous fish species fed with high levels of dietary carbohydrates that either reveal high triacylglycerol hepatic levels (Viegas et al., 2016; Kamalam et al., 2017) or export that excess energy to extrahepatic tissues like the muscle or perivisceral fat (Kamalam et al., 2017; Viegas et al., 2019). Such possible metabolic fate of dietary glycerol could also explain the negative effect of dietary glycerol on nitrogen efficiency detected as previously measured during the dietary trial (Magnoni et al., unpublished data).

## Conclusion

Overall, our data characterize, for the first time in fish species, all the genes involved in glycerol metabolism in rainbow trout and their expression during ontogenesis. Many of these genes were regulated by the nutritional status from the first feeding stage up to the juvenile stage. However, the incorporation of increased levels of glycerol (up to 5%) did not modify the mRNA levels for the glycerol genes.

## Data Availability Statement

The authors acknowledge that the data presented in this study must be deposited and made publicly available in an acceptable repository, prior to publication. Frontiers cannot accept a manuscript that does not adhere to our open data policies.

## Ethics Statement

The study was conducted under the supervision of accredited experts in laboratory animal science by the Portuguese Veterinary Authority (1005/92, DGV-Portugal, following FELASA category C recommendations), according to the guidelines on the protection of animals used for scientific purposes from the European directive 2010/63/UE.

## Author Contributions

SP and IV contributed to the conceptualization and supervision. SP, EP-J, and LM contributed to the formal analysis. IV contributed to the funding acquisition, project administration, and resources. EP-J, EG, SP, LM, LJM, and IV contributed to the investigation. EP-J, EG, and SP contributed to the methodology. SP, LM, LJM, and IV contributed to the validation. EP-J and SP contributed to the writing of the original draft. LJM, LM, and IV contributed to the writing – review and editing. All authors contributed to the article and approved the submitted version.

## Conflict of Interest

The authors declare that the research was conducted in the absence of any commercial or financial relationships that could be construed as a potential conflict of interest.

## Footnotes

1 http://www.ensembl.org

## Abbreviations

Aqp9: aquaporin 9
*aqp9b*: aquaporin 9b gene
DHAP: dihydroxyacetone phosphate
G3P: glycerol-3-phosphate
*gk*: glycerol kinase gene
Gk: glycerol kinase
*gpd*: glycerol-3-phosphate dehydrogenase gene
Gpd1: cytosolic glycerol-3-phosphate dehydrogenase 1
*pgp*: glycerol-3-phosphate phosphatase gene
Pgp: glycerol-3-phosphate phosphatase.
